# Pregnancy and Gastric Cancer: A Narrative Review

**DOI:** 10.3390/diagnostics13111909

**Published:** 2023-05-29

**Authors:** Adrian Constantin, Roxana Constantin, Florin Achim, Bogdan Socea, Dragos Predescu

**Affiliations:** 1 Department of Esophageal and General Surgery, Sf. Maria Clinical Hospital Bucharest, 011192 Bucharest, Romania; 2 Faculty of Medicine, Carol Davila University of Medicine and Pharmacy Bucharest, 050474 Bucharest, Romania; 3 Department of Obstetrics and Gynecology, Sanador Hospital, 010991 Bucharest, Romania; 4 Department of Surgery, Sf. Pantelimon Emergency Clinical Hospital, 021659 Bucharest, Romania

**Keywords:** pregnancy, gastric cancer, multimodal therapy

## Abstract

Cases of digestive cancers diagnosed during pregnancy are rare. The increasing prevalence of pregnancy in women aged 30–39 years (and not exceptionally 40–49 years) could explain the frequent co-occurrence of cancers and pregnancy. The diagnosis of digestive cancers in pregnancy is difficult due to the overlap between neoplasm symptomatology and the clinical picture of pregnancy. A paraclinical evaluation may also be difficult depending on the trimester of the pregnancy. Diagnosis is also delayed by practitioners’ hesitation to use invasive investigations (imaging, endoscopy, etc.) due to fetal safety concerns. Therefore, digestive cancers are often diagnosed during pregnancy in advanced stages, where complications such as occlusions, perforations, and cachexia have already arisen. In this review, we highlight the epidemiology, clinical aspects, paraclinical evaluation, and particularities of the diagnosis and treatment of gastric cancer during pregnancy.

## 1. Introduction

Gastric neoplasia in young adults is rarely reported in the literature, and cases of pregnancy-associated stomach cancer are even rarer [1,2,3,4,5]. Although it was once the most common neoplasia in adults, there has been a constant decrease in the incidence of gastric cancer globally, now ranking sixth in the distribution of cancers and fifth in cancer mortality in women [6,7,8].

Gastric cancer remains one of the most common types of cancer, with very specific geographical, ethnic, and socioeconomic differences in incidence [9]. More than 70% of gastric cancer cases occur in developing countries, where the majority of patients are from East Asia [10].

Pregnancy-associated gastric cancer, defined as a diagnosis of gastric cancer during pregnancy or up to one year after delivery, is estimated to complicate 0.026–0.1% of all pregnancies [10].

In pregnancy, gastric cancer is staged according to the American Joint Committee on Cancer/Union for International Cancer Control TNM staging system, which is based on tumor size (T), lymph node invasion (N), and metastatic disease (M). The distribution in the general population is 21.6% for stage I, 22.3% for stage II, 44.0% for stage III, and 12.1% for stage IV [11].

## 2. Search Strategy

This review is based on an analysis of data from relevant studies and articles published in the last 23 years (corresponding to the period 2000–2022) identified in Embase (Excerpta Medica Database), PubMed Central (PMC), Cochrane Library, and MEDLINE Complete (EBSCO). With respect to inclusion criteria, sources written in English, including book chapters, studies, study updates, case presentations, original articles, and reviews related to cancer and pregnancy—particularly for cancers of the digestive tract and especially stomach neoplasia—were included. The search strategy was based on keywords and phrases used in search engines, namely the following terms: “cancer and pregnancy”, “digestive cancers and pregnancy”, “diagnosis of cancer in pregnancy”, and “treatment of cancer in pregnancy”. Following the search, 468 book chapters, articles, and studies on the issue were identified. For selection based on topic, we used “advanced option” and introduced additional criteria: “gastric cancer during pregnancy”, “diagnosis of cancer during pregnancy”, and “treatment of gastric cancer during pregnancy”. This allowed for the “search history” to be displayed and for the combination of the individual searches using the Boolean operators “AND” and “OR.” Using this method, parentheses were automatically placed around each set of terms to maintain the logical structure of the search. From this, 43 scientific publications strictly concerning gastric cancer during pregnancy were identified. We also included a series of articles from the initial reference lists for additional information considered relevant to the problem. Two authors (A.C. and D.P.) independently selected articles they deemed relevant, with a preference for articles from high-ranking journals written in English. The decision to select an item was made by mutual agreement. The number of citations of the respective work was an important selection criterion. An assessment time was set for each article: 15 min for clinical cases and 30–60 min for reviews, original articles, and book chapters. Differences were discussed, and if consensus could not be reached between the two reviewers, we requested the consultation and recommendation of a third reviewer (F.A.). We excluded unpublished data from abstracts contained in volumes from various congresses or conferences, as well as papers that were not in English.

## 3. Epidemiology and Etiology

The distribution of cancer incidence in females highlights an important geographic variation, where some regions have an extremely high rate, e.g., Japan and Korea [12,13] (27–28.6/100,000), while others have an average (Costa Rica and Brazil) or low rate (North America, Australia, and New Zealand) [14]. Less than 10% of gastric cancers are diagnosed under the age of 45, the maximum possible age for pregnancy, while for a maximum age of 36, the incidence of gastric cancer is under 5%. The prevalence of gastric cancer in pregnant women is 0.026–0.1% [15]. In the Western literature, the total number of reported cases of pregnancy-associated gastric cancer is 168, of which 137 were from Japan, and 31 were from Western countries [16]. There is no specific incidence of gastric cancer in pregnant women when compared to that in the general population, and even if the definition of gastric neoplasia is moved towards the eso-gastric junction, no cases with this localization have been reported in pregnant women [17].

A review from China carried out by Zeng and Zhou in 2015 on 65 pregnant patients with gastric cancer highlighted an advanced stage in all situations [18]. In Japan, out of 136 cases of pregnant women with gastric cancer evaluated by Sakamoto et al. in 2009, 92.5% were at advanced stages, and only 43.5% benefited from resection [19]. In the same study, survival was 18% at one year and 15.1% at two years. In a 2016 study, Jeong Song et al. reported that the poor prognosis of pregnancy-associated gastric cancer was due to the advanced stage at diagnosis [20]. The authors concluded that early diagnosis and surgical treatment are the only factors that can improve patient outcomes.

Etiologically, the risk of pregnant women developing gastric cancer is identical to that of the general population.

Histologically, gastric cancer has two forms, intestinal and diffuse, where each type is characterized by an etiological mechanism [21].

The intestinal type is etiologically correlated with environmental elements, especially diet, lifestyle, and Helicobacter pylori infection, hence the considerable variability in the geographical incidence of the disease.

The diffuse type is most often associated with genetic factors. Some groups of patients are classified as high risk due to pre-existing precancerous conditions.

## 4. Diet and Eating Habits

The link between gastric cancer and diet is complex and difficult to demonstrate. Numerous studies have revealed an association between an increased risk of gastric cancer and salt consumption as, in Japan, a diet rich in smoked foods, especially fish and very salty pickles, are carcinogenic factors. Food industrialization with numerous preservatives (n-nitroso), dyes, and additives has increased the risk; a slight increase also occurs in consumers of red meat. A diet rich in fruits and vegetables lowers the risk of gastric cancer, with evidence being found in vegetarians [22]. Alcohol consumption does not seem to play an important role in the etiology of gastric cancer. On the other hand, smoking increases this risk by two times compared to non-smokers; if it is associated with an H. pylori infection, the risk increases by ten times [23].

The association between H. pylori infection and gastric cancer is well-established [24]. Currently, H. pylori infection is considered the most important trigger of the carcinogenic sequence, initiating the progression of chronic gastritis–atrophic gastritis–intestinal metaplasia–dysplasia–adenocarcinoma [25]. The risk of gastric cancer is 2.1–16.7 higher in H. pylori-seropositive patients than in seronegative individuals, especially for those carrying a subtype with increased inflammatory properties (H. pylori cagA) [26].

Various gastric pathologies can be precursors of neoplasia, including gastric dysplasia, various benign tumors (adenomatous polyps (10% risk per year), hyperplastic polyps with an adenomatous component (1% risk), and hamartomatous polyps (risk below 1%)), Biermer’s anemia, Menetrier’s disease, and ulcers. The risk of neoplasia in polyps is related to the size and the villous component. The higher the number, the higher the risk. The following situations may exist: single polyps or multiple (2–10) or diffuse polyposis (diffuse polyps, familial hamartomatosis).

The etiology of gastric cancer can be hereditary in young adults (average age of 38 years). Hence, there is a possibility of gastric cancer overlapping with pregnancy. A previous review of the literature found that the existence of a first-degree relative with gastric neoplasia increases the risk of gastric cancer by 2–3 times [27].

Syndromes that predispose individuals to the occurrence of gastric cancer include hereditary diffuse gastric cancer (autosomal dominant pathology), non-polyposis colorectal cancer, familial adenomatous polyposis, and syndromes such as Cowden or Peutz–Jeghers [28].

Molecular studies have confirmed that genetic alteration is essential in the initiation and support of the carcinogenic process. Similar to carcinogenesis in colorectal cancer, these alterations occur at the level of suppressor genes, which inhibit tumor development by regulating growth and differentiation, or oncogenes, whose activation stimulates tumor transformation. The alteration is bivalent and synchronous, causing the evolution towards cancer.

One example is the e-cadherin mutation, which results in the dysregulation of gene expression, leading to a loss of cell adhesion with increased invasiveness [29]. This mechanism is most common in young adults, including pregnant women. Microsatellite instability, considered a variant of altered DNA replication, has been identified in approximately 20–30% of gastric cancers, especially in cases with a genetic predisposition. The alteration of genetic material with a secondary carcinogenetic effect is involved in various hereditary syndromes. In familial adenomatous polyposis, the aPC gene is involved; the inactivation of the gene at the level of chromosome 5q is responsible for about 20% of diffuse gastric neoplasias. The PTEN gene in the Cowden (chromosome 10p) and Peutz–Jeghers (chromosome 19p) syndromes initiates the polyp–cancer sequence. Among the suppressor genes, the most studied is TP53; however, other genes are also involved due to chromosomal losses (loss of heterozygosity lOH) located at 1p, 5q, 7q, 11p, 13q, 17p, and 18p [30,31].

In pregnancy, the established etiological factors of gastric cancer remain up-to-date, with the mention that the physiological features of pregnancy can participate in neoplastic etiopathogenesis. Isobe et al. demonstrated that estrogens, which increase during pregnancy, favor the growth of diffuse-type adenocarcinoma, and the immunosuppressive influence of pregnancy can be an additional factor in the development of the cancer [32,33].

## 5. Histology

**Figure 1:**
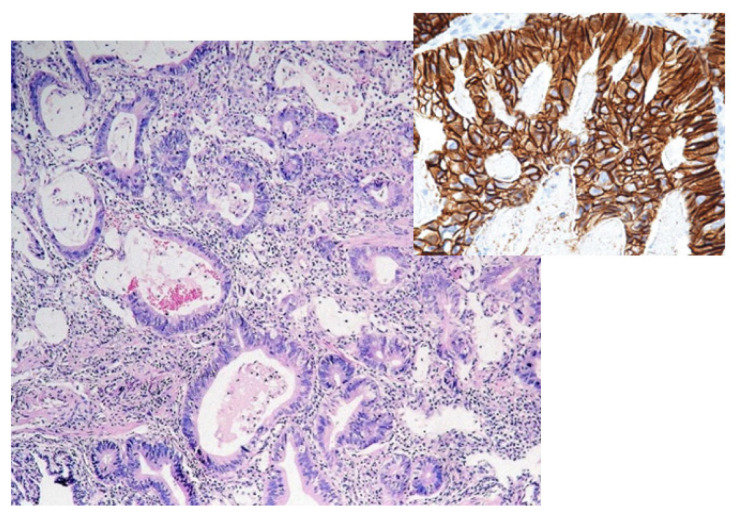
Histopathological aspects of intestinal gastric adenocarcinoma (left; HE, ×40). Immunohistochemical examination of gastric adenocarcinoma with the overexpression of human epidermal growth factor receptor 2 (right; ×400 HER2 positive 3+).

**Figure 2:**
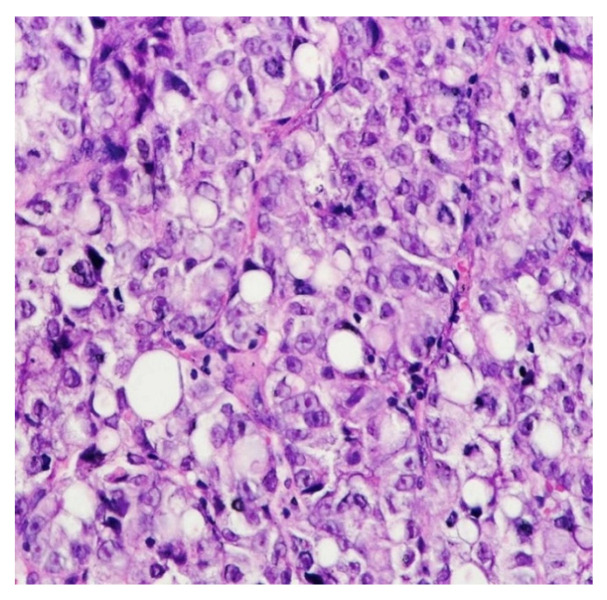
Histopathological aspects: gastric signet ring cell adenocarcinoma (HE, ×40).

In the general population, the main histological subtypes of gastric carcinoma are the intestinal (54%) and diffuse (32%) types; the remaining 10–12% are considered rare and indeterminate (Figure 1). In pregnant women, the most common type is diffuse [34]. The main patterns of histological development (WHO 2010) are tubular and papillary but also mucinous, including “signet ring” or rare variants (adenosquamous carcinoma, squamous carcinoma, hepatoid adenocarcinoma, carcinoma with lymphoid stroma, choriocarcinoma, etc.) (Figure 2) [35]. The two well-known types of tumor development with a prognostic and treatment role are early gastric carcinoma and advanced cancer.

The early type is defined as invasion limited to the mucosa/submucosa regardless of tumor size, with or without lymph node metastases (the Japanese description of early tumors describes four main types, while the Paris classification describes a variant with six types [36].

## 6. Diagnosis

The clinical component of diagnosis is difficult; the initial symptomatology is non-specific and unfolds over the years, during which time it is attributed to other pathologies. Hence, framing the concomitance of pregnancy and gastric cancer as a function of the time criterion is difficult, with some extending this interval up to two years from the moment of birth. Most early cancers are asymptomatic or have non-specific symptoms (non-ulcer dyspepsia, peptic ulcer). The consequence will be a delay in diagnosis and treatment. The diagnosis of advanced cancers becomes obvious due to the complications of the disease.

Nausea and vomiting, pseudo-ulcer pain (without ulcer periodicity or unrelated to food), anorexia, and change in appetite, although non-specific, are the most common symptoms of gastric cancer.

There are several factors that cause nausea and vomiting in pregnancy (the main symptoms of gastric cancer). Symptoms often begin a few weeks into the first trimester, then peak between 10 and 16 weeks of gestation and remit by 20 weeks; however, up to 10% of women may be asymptomatic until 22 weeks. The peak of vomiting occurs simultaneously with the peak of hCG production at 12–14 weeks. Another hormone related to this clinical picture is prostaglandin E2 (PGE2), which affects gastric smooth muscle. The highest level of PGE2 in pregnancy occurs between 9 and 12 weeks [37]. Hyperemesis gravidarum is a severe form of nausea and vomiting associated with the loss of more than 5% of pre-pregnancy weight, dehydration, and electrolyte imbalances. It usually starts before the 22nd week of pregnancy, affects 0.3–2.0% of pregnancies, and sometimes requires hospitalization [38]. A Canadian population-based cohort study by Fell et al. reported an increased risk of hyperemesis gravidarum associated with hyperthyroidism, psychiatric disease, previous molar pregnancy, diabetes, and preexisting asthma [39].

Currently, three major etiologies have been described in the literature. First, high levels of HCG can have a stimulating effect on the secretory process in the upper gastrointestinal tract. In addition, the production of thyroid-binding globulin increases under estrogenic stimulation, leading to a decrease in free thyroxine (T4). The transient decrease in free T4 causes thyroid stimulation, and the patient may develop transient gestational thyrotoxicosis, which leads to vomiting. Second, HCG is similar to thyroid-stimulating hormone (TSH) and possibly causes hyperemesis by stimulating the TSH receptor [40].

Third, there is a negative relationship between prolactin levels and nausea/vomiting, while estrogens show a positive relationship. Therefore, higher estrogen levels during pregnancy may increase the risk of hyperemesis gravidarum [41].

Most of the time, it requires a diagnosis of exclusion. The condition is usually accompanied by hyponatremia, hypokalemia, low serum urea, increased hematocrit, metabolic hypochloremia, alkalosis, and ketonuria. Liver enzyme levels may be elevated in 50% of cases. Patients are dehydrated and suffer from food intolerance and weight loss due to prolonged vomiting [42]. In a study by Song, 25% of patients had abdominal pain, 20% had nausea and vomiting, and the rest had bleeding and metastatic symptoms [20]. Cift et al. recommended a radiological examination in pregnant women who complained of epigastric pain, refractory nausea, and vomiting that manifest at a gestational age greater than 16 weeks [43].

Upper digestive bleeding (described in 20% of gastric cancer cases) can be attributed to Mallory–Weiss syndrome, the most common cause of hematemesis in pregnancy [44].

However, there are no protocols in the literature regarding the best time to perform an endoscopy for pregnant women with nausea and vomiting in the first trimester of pregnancy when, moreover, the level of hCG and PGE2 reaches its peak. The indication for screening for gastric malignancy in pregnant women remains similar to that for the general population.

A clinical examination is, therefore, not able to specify the diagnosis, although there are enough elements that should raise the suspicion of neoplasia.

In the case of an acute complication (perforation or bleeding), specific clinical signs appear, with immediate severe consequences for the mother and the fetus. Emergency surgical treatment is required in the context of an acute abdomen tear by tumor perforation or signs of significant hemorrhage (hematemesis/melena).

### 6.1. Paraclinical Diagnosis

Serological evaluation is mandatory but lacks diagnostic specificity. Changes in the hemogram and biochemical tests, with respect to tumor markers, are characteristic of any other advanced neoplasia of the digestive tract. Physiological changes specific to pregnancy should not be ignored in order to correctly interpret the results of the investigations.

Pregnancy is characterized by a state of hemodilution induced by volume expansion due to salt and water retention. Consequently, there is a decrease in hemoglobin and albumin levels. Platelet levels may drop but usually remain within the normal range. Alkaline phosphatase may show a three- to four-fold increase due to placental production. Aspartate aminotransferase, alanine aminotransferase (ALT), γ-glutamyl transferase (GGT), bilirubin, and prothrombin time (PT) remain within normal limits. However, coagulation factors are affected by pregnancy, with a slight decrease in antithrombin III, protein C, and protein S and an increase in factors I to X, XII, and fibrinogen, which favor a pro-coagulant state [45]. Increased levels of hormones, such as progesterone, contribute to delayed gastric emptying. Gastric acidity is increased due to the increased production of gastrin by the placenta [46].

### 6.2. Upper Digestive Endoscopy

**Figure 3:**
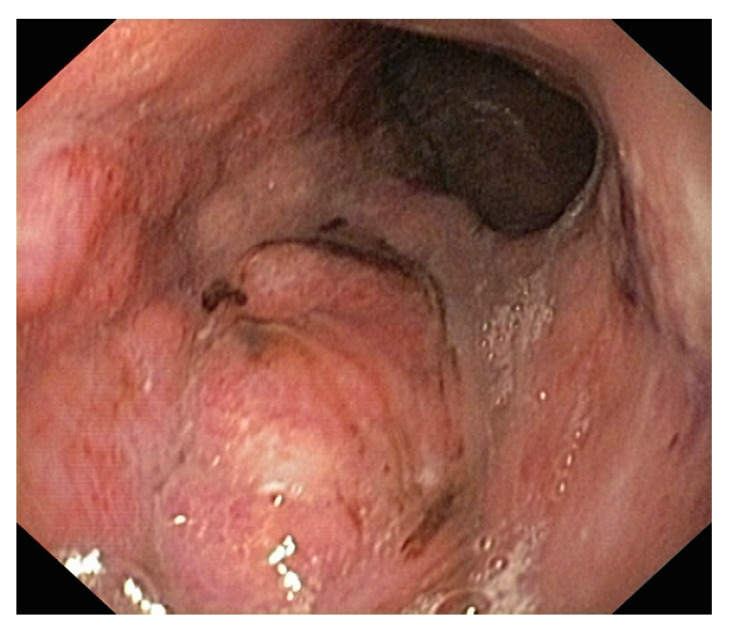
Endoscopic aspect: large gastric adenocarcinoma.

Endoscopic evaluation is essential to the diagnosis of gastric cancer (Figure 3). Essential for the endoscopic evaluation is the type of indication: elective or emergency, which affects the preparation of the pregnant woman, requiring specific steps to minimize risks. Upper digestive bleeding, especially in its severe forms, requires endoscopy and often concomitant therapeutic interventions. Some rules are mandatory: NPO 6–7 h before the investigation, the mother’s left lateral decubitus position during endoscopy for optimal uterine and fetal perfusion, nasogastric tube, the administration of oxygen to the pregnant woman and oximetry, blood pressure control, cardiac fetal activity monitoring, and a peripheral venous line [47]. Teratogenic or abortion risks are small and usually encountered in the first trimester. Administered medication should be limited as much as possible. If necessary, sedation should be performed with meperidine or fentanyl and not with diazepam/midazolam due to the minimal effect on the fetus [48].

In general, the studies carried out on pregnant women who had an esophagogastroduodenoscopy with various indications have not confirmed risks for the fetus, with over 95% having normal newborns and a fetal status without complications [49,50]. Whenever a possible neoplasia is suspected, endoscopy must be recommended without hesitation, and the technique must be associated with the biopsy of the tumor. Unfortunately, the mimetic nature of malignancy with symptoms that are common during pregnancy causes the diagnosis to be delayed, and often an advanced-stage neoplasia is revealed. Modern endoscopy techniques (narrow banding, magnification, confocal, chromoendoscopy, etc.) associated with EUS can be used to clarify the diagnosis of early cancers.

A review of the literature reported only six patients with early-stage disease [51,52,53]. In the USA, over 12,000 pregnant women have an indication for endoscopy annually [54]. The procedure is not without risks. The sedation used during the maneuver alone can cause maternal hypotension, maternal hypoxia, arrhythmias, aspiration, and fetal hypoxia. The fetus can be exposed to teratogenic substances, and the risk of premature birth is increased [55]. In 2017, Ludvigsson et al. rigorously analyzed a group of 3052 pregnant women who benefited from undergoing an endoscopy [56]. The relative risk of major congenital malformations after endoscopy vs. no endoscopy during pregnancy was 0.98, with a narrow 95% confidence interval (0.82–1.19) [56].

Endoscopy allows for therapeutic maneuvers to be carried out [57]. Epinephrine injection, thermocoagulation, and electrocoagulation have been shown to be successful during pregnancy. Epinephrine can reduce blood flow to the fetus. However, no adverse effects have been reported in the literature [58]. In electrocoagulation, the amniotic fluid can conduct an electrical current to the fetus; thus, there should be a grounding pad placed away from the uterus, and bipolar electrocautery should be used to minimize the risk. However, there are limited data on hemostasis for non-variceal bleeding in pregnant women, the indications and technique being based on expert opinion from non-pregnant patients [44].

### 6.3. Abdominal Ultrasound (AU)

AU is the first choice for imaging evaluation in pregnancy because it can be performed quickly and safely. AU involves the use of sound waves and is not associated with any exposure to ionizing radiation for the mother and fetus. There have been no reports of adverse fetal effects for diagnostic ultrasonography procedures, including Doppler imaging [59]. The role of AU is essential in advanced cases in the appreciation of the N and M indexes; however, in the hands of an experienced sonographer, the pathology of the gastric wall can be identified (T index), especially after filling the stomach with water before exploration [60]. The limits of AU are obvious, however, for the detection of early gastric cancer. There is a permanent interest in improving the information provided through this examination. Gastric filling ultrasonography involves the oral administration of a contrast substance to determine the disappearance of the gastric gas content level, transforming the gastric cavity into a homogenous ultrasound image similar to parenchymal organs. The gastric wall can be assessed in detail, providing accurate information on the location, number, and depth of the tumors. A recent meta-analysis (Zhang DN et al., 2021) showed an accuracy of gastric filling ultrasonography for gastric cancer of up to 94% [61]. The role of this method in assessing the intraparietal depth of gastric cancers has been reported in the literature; however, the results are inhomogeneous, and studies on large groups of patients are lacking [62].

The method has the advantage of being safe and feasible, with good patient compliance [63]. However, many studies reveal that the results are not statistically significant and are rarely used for gastric cancer in pregnancy [64,65].

Radiological tests, such as barium swallow, CT, and MRI, are generally rarely indicated and used only where there is no other diagnostic resource or in the face of major emergencies.

Abdominal X-ray or barium swallow are rarely used during pregnancy, these being replaced by more complex radiological explorations, which through the details provided, can justify the risk of radiation exposure.

### 6.4. Computed Tomography (CT)

**Figure 4:**
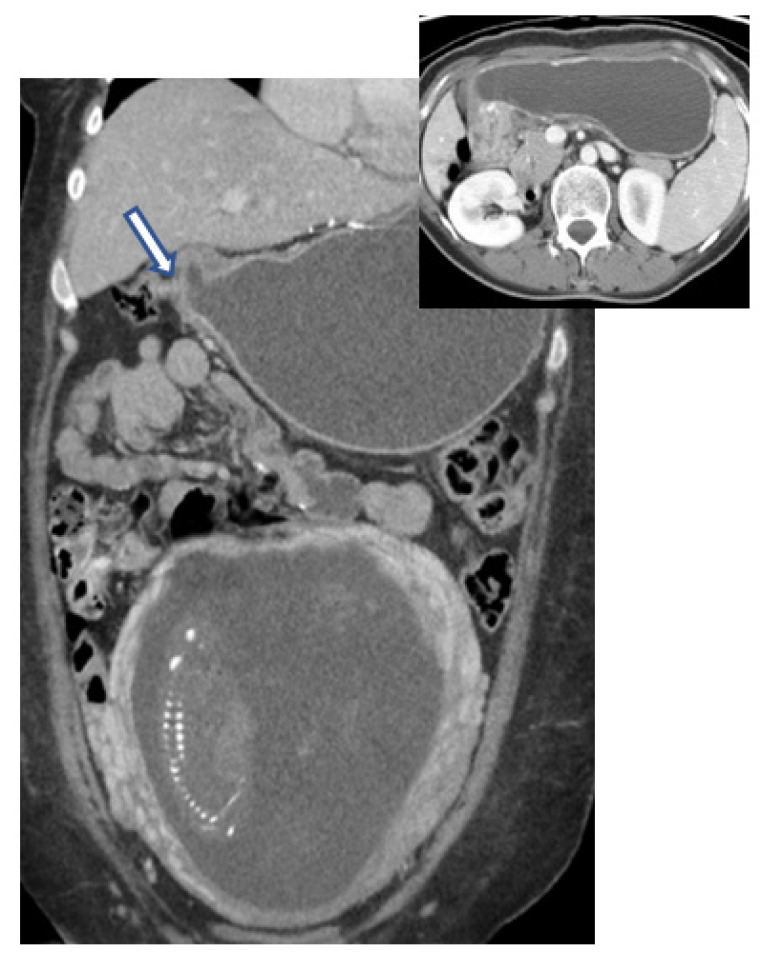
Computed tomography imaging of a pregnant woman (gestational age 16 weeks) with pyloric obstruction (arrow) caused by gastric adenocarcinoma.

If CT can be useful for a diagnosis, especially of a major complication, or as a therapeutic guide, the practitioner should not hesitate to use it, as the life of the mother prevails over the fetus's prognosis. What can CT bring in terms of evaluating neoplasia in pregnancy? Firstly, the method allows for a three-dimensional evaluation of the relationships between the tumor and adjacent structures. Secondly, the assessment and demonstration of complications that are not apparent or lead to a poor prognosis of the mother in the absence of a clear or obvious diagnosis put her life in immediate danger, especially in the absence of a quick and effective treatment (perforations, pyloric obstruction) (Figure 4). Thirdly, CT is used for disease staging. After initial enthusiasm, in which CT was credited with an accuracy of 85–90% in the assessment of the T index from TNM staging, rigorous studies have shown a much lower rate, between 50 and 70%, depending on the stage of the cancer [66,67].

The overstaging of the T index and assessment of the N index is the most common problem (specificity in the evaluation of lymph nodes is only 45%). The essential role of CT is in the assessment of the M index, identifying liver metastases with an accuracy of 85% and a specificity of 97%. The data are relatively similar to those obtained using MRI [68].

Over the last 40 years, CT has been avoided in pregnant women due to its teratogenic or carcinogenic effects on the fetus. Now, the use of spiral CT has reduced radiation exposure time (17–19 s for the abdomen or pelvis with 1.25 mm slices).

For this reason, CT has been reconsidered in pregnant women [69]. The potential effects of exposure to ionizing radiation on the fetus include an increased risk of malformations, neurodevelopmental disorders, and carcinogenesis. The risk of malformations, developmental abnormalities, or intrauterine growth restrictions depends on the time and dose of ionizing radiation exposure [70]. The fetus is most sensitive during the period of major organogenesis and early fetal development (2–15 weeks gestation). Doses above 100 mGy of radiation are believed to induce malformations based on experimental animal data [71].

Risk of fetal malformations, intrauterine growth restriction, or abortion has not been reported after exposure to radiation below 50 mGy [72]. Similarly, for neurodevelopmental effects, the most sensitive period is 8–15 weeks of gestation and in association with radiation doses of at least 100 mGy [71]. The risk of intellectual disability implies exposure of at least 60–310 mGy between 8 and 15 weeks of gestation [73]. Abdominal and pelvic CT scans are associated with increased radiation exposure compared to head or chest scanning; however, fetal dose exposure from an abdominal and pelvic CT scan is significantly lower than the hazard level. Fetal ionizing radiation doses for common procedures are approximately 0.01 mGy for a chest X-ray, 0.66 mGy for a chest CT, 3.0 mGy for an abdominal X-ray, up to 35 mGy for an abdominal CT, and up to 50 mGy for a pelvic CT (Table 1) [74]. Radiation exposure from CT also varies depending on the number and distance between sections. During CT, the exposure of the pelvis to radiation can be reduced to approximately 13 mGy by using dose reduction techniques, if appropriate, while still obtaining acceptable semiological data for certain diagnoses [75]. By comparison, any fetus is estimated to be exposed to about 1 mGy of background radiation during a normal pregnancy. There is no dose threshold related to the occurrence of childhood cancer, from which increasing radiation doses would cause neoplasia.

However, the relationship between cancer and low-dose radiation exposure is controversial, with data in the literature being inconsistent on this issue [76]. A fetal exposure of 10–20 mGy can increase the risk of leukemia 1.5–2-fold above a background incidence of about 1 in 3000. The long-term monitoring of children exposed to CT radiation in utero is not currently recommended [75]. Exposure to high doses (above 1000 mGy or 1Gy) early in embryogenesis is probably lethal to the embryo [70]. However, such doses are not used in diagnostic imaging. In humans, fetal growth restriction, microcephaly, and intellectual disabilities are the most common adverse effects of high-dose radiation exposure, following the evaluation of data from atomic bomb survivors [75]. Abortion is not mandatory after exposure to radiation if multiple imaging exams using ionizing radiation have been performed; the total dose received by the fetus needs to be calculated by a physician to aid in risk assessment [76].

Contrast can be administered if needed for additional diagnostic details. Oral contrast agents are not absorbed by the patient and are not harmful. Intravenous iodinated contrast substances can cross the placenta and enter fetal circulation or pass directly into the amniotic fluid [77]. However, animal studies have not reported teratogenic effects or thyroid disorders in infants exposed to water-soluble iodinated contrast [78,79]. Only a very small percentage of the iodinated substance is excreted in breast milk and absorbed by the infant; therefore, it is considered safe to continue breastfeeding after the administration of an iodinated contrast agent. External sources of ionizing radiation cannot affect breast milk. Regarding the maternal risks of exposure to ionizing radiation, the main concern relates to breast tissue in pregnant women during chest CT. Examination in low voltage mode can be an acceptable decision [59].

**Table 1:** Fetal radiation doses of the most frequently used imaging methods in oncology (adapted from ACOG Committee Opinion: Guidelines for Diagnostic Imaging During Pregnancy and Lactation) [80].

Fetal Dose (mGys)	0	0.001–0.1	0.1–1.0	1.0–10	10–50
Imaging tests	US MRI	X-ray (head, chest, extremity) Mammography CT (head and neck) Cervical spine radiography	X-ray (abdomen, pelvis) Lumbar spine radiography CT (chest)	Abdominal CT Technetium-99 m bone scintigraphy	CT (pelvis) PET-CT FDG

### 6.5. MRI

MRI is an alternative used in pregnant women instead of CT for stage assessment, having the advantage of providing information and complex topographic details (3D). It allows for the identification of tumor recurrence from postoperative fibrosis. MRI has similar limitations to CT regarding staging (N and M) but with much higher costs [81].

Sohn et al. suggest that there are no differences between MRI and spiral CT scan [82]. The landmarks to follow for an MRI are the same as those for a CT scan. The major disadvantage is the presence of artifacts induced by enteral peristaltic movements and respiratory movements (the scan lasts between 5 and 10 min). Endoscopic MRI is at least as effective in assessing parietal tumor invasion as EUS. Some studies have found a significant improvement in the accuracy of T-score assessment, with a sensitivity of 100% and a specificity of 86% [83]. There are concerns regarding the safety of the method in the case of pregnant women due to the possible teratogenic effects of magnetic fields and potential acoustic damage to the fetus. The in vivo animal studies conducted by Heinrichs et al. and Tyndall et al. showed the presence of malformations following exposure to magnetic fields (e.g., eye malformations) and the death of embryos or their abnormalities when the procedure was performed during the period of organogenesis [84,85,86]. As a result, although similar effects have not been proven in humans, the indications of the National Radiological Protection Council in Great Britain recommend that “it is prudent to avoid MRI in pregnant women during the first three months of pregnancy”. Possible acoustic effects induced in the fetus by the high sound level during MRI are more theoretical [87]. Regarding the contrast, no teratogenic effects have been reported [88,89].

However, there is uncertainty regarding the use of gadolinium during pregnancy. Gadolinium is water soluble and can cross the placenta into the fetal circulation and amniotic fluid [90]. Free gadolinium is toxic and is therefore administered in a chelated (bound) form [75]. In animal studies, gadolinium has been found to be teratogenic at high levels and in repeated doses [89]. This is thought to be due to dissociation from the chelating agent and prolonged exposure as the contrast remains in the amniotic fluid and is swallowed by the fetus before re-entering the fetal circulation [75]. The longer the presence of the gadolinium chelate molecule in the amniotic fluid, the higher the risk of gadolinium dissociation from its ligand and, hence, the greater the risk of free gadolinium ions harming the fetus [90]. A recent large retrospective study from Canada identified all births greater than 20 weeks’ gestation from 2003 to 2015 and compared cases with first-trimester MRI exposure to cases not exposed to this exam [91]. There was no significant increase in the risk of neonatal death, congenital anomalies, neoplasm, or hearing loss in MRI-exposed cases. However, higher rates of rheumatologic, inflammatory, or infiltrative skin disease by four years of age have been reported in some cases with MRI exposure [91]. Therefore, given these concerns about gadolinium, contrast-enhanced MRI in pregnant women is performed only when it is crucial for diagnosis [77]. MRI should be performed without gadolinium or delayed until postpartum if possible. In breastfeeding women, MRI can be performed with gadolinium because its water solubility limits excretion in breast milk. Less than 0.04% of a standard dose of gadolinium is excreted in milk in the first 24 h after administration. The infant will absorb less than 1% through the gastrointestinal tract [75]. Therefore, breastfeeding can be safely continued [92]. In conclusion, in the first trimester of pregnancy, MRI is preferable to any other investigative method using ionizing radiation.

### 6.6. PET-CT

PET-CT is not essential for the management of gastric cancer in pregnancy. On the other hand, pretherapeutic staging defines the oncological approach and contributes to choosing the best option for both the mother and fetus in a shared decisional manner. Therefore, it is crucial to assess the stage of the disease by means of highly accurate imaging tools to evaluate both the status of lymph nodes and metastases, where PET-CT is essential. PET-CT is not currently used for evaluation in pregnant women; however, for selected cases, it can be used with some specific recommendations (a reduction in the 18F-FDG dose, the use of the 3D technique, which will allow the reduction in the 18F-FDG dose, good hydration, the attenuation of CT voltage, etc.). The role of PET-CT in the assessment of gastric cancer is to identify the tumor site and assess the metastases. Technological development has allowed cumulative fetal radiation doses during and after PET-CT to be at safe levels (between 1 and 2 cGy), comparable to or even lower than spiral CT [93,94,95]. (18F)-Fluorine-(2)-deoxy-glucose ((18F)-FDG) positron emission tomography/computed tomography (PET/CT) imaging is a gold standard in the evaluation of oncological patients; with certain precautions, this goal is also kept in pregnant women [96,97,98]. Although some small case series studies have estimated fetal radiation exposure in pregnant women to be well below the threshold dose for both deterministic and stochastic effects, the indication for (18F)-FDG PET/CT remains a matter of debate [99]. The CT component contributes 8.4–30.8% of the total absorbed dose, while the PET component contributes 70–91.6% in evaluation by this technique [100]. In a study published in 2015, Sawatzke et al. used PET/CT in mice to explore the placental–fetal transport of (18F)-FDG and developed a kinetic model of glucose transport from the placenta to the fetus [101]. They revealed that the placental avidity for glucose was greater than any other tissue examined in the maternal or fetal body and that the fetal uptake of FDG was weight dependent. Thus, the placenta and fetus have a high-glucose metabolic system, accounting for a quarter of the total FDG dose administered, with a potential negative impact. In 2017, Gill et al. conducted a study of pregnant patients who underwent 18F-FDG PET/CT using dosimetry data [102]. All fetal 18F-FDG doses were below the threshold for deterministic radiation risks. The effects of radiation on the fetus, such as intellectual disability or malformation, could occur above a threshold dose of 100–200 mGy. The effects of radiation depend on the gestational age, and radiation in the first week after conception will lead to implantation failure, while malformations can occur if the threshold is reached during organogenesis [103,104].

## 7. Treatment

The therapeutic plan for the treatment of gastric cancer in pregnancy should respect one axiom: treatment as quickly as possible for the mother but after delivery as soon as possible for the baby. The general principle—the life of the mother prevails when both cannot be preserved—is challenging. The family should not be excluded from these decisions, nor should legal, ethical, religious, or personal/emotional issues be neglected.

Optimal management requires a multidisciplinary approach (including an oncologist, obstetrician, surgeon, anesthetist, gastroenterologist, radiologist, and neonatologist), which will establish the sequence of the therapy. Psychological supportive therapy should not be neglected because the mother’s decision is crucial. She may do everything so the therapy will not be harmful to the fetus. The mother’s decision is very strongly linked to fetal survival, sometimes with her sacrifice.

### 7.1. Chemotherapy

With respect to therapy with cytotoxic agents, there is the issue of the efficiency of chemotherapy related to the physiological changes in pregnancy (volume redistribution, altered hepatic clearance, increased renal elimination by decreasing binding protein, decreased albumin levels with increased serum levels of unrelated drugs, etc.). However, at least at this moment, there are no specific recommendations different than those in the basic population regarding dosage. These doses will undergo changes along with the weight gain and gestational age of the pregnant woman [105,106,107].

If we have some prediction of the oncological therapy in the mother, the question arises what happens at the fetal level, and what is the impact of the chemotherapy on pregnancy? What are, if any, the immediate and possibly remote fetal consequences to the newborn and, later, on the child and the adult? Regarding the immediate effects, the most commonly reported are the risk of spontaneous abortion or premature birth, fetal death, visceral toxicity, teratogenesis, and malformations [108]. Among the most feared long-term complications are teratogenic and mutagenic effects. The risk of carcinogenesis over time appears to be critical. The fetal toxic effect would be the consequence of the cytostatic crossing the maternal–fetal barrier. The fetal liver will metabolize, and the kidneys will eliminate the toxins into the amniotic fluid, from where it can be swallowed by the fetus and reabsorbed in the gastrointestinal tract. The most studied teratogens are anthracyclines, being found in the placenta, umbilical cord, and fetal tissues, while two other studies on doxorubicin did not identify the drug in the amniotic fluid [109,110,111,112].

In order to reduce these effects, a reduction in doses has been proposed; however, taking into account the pharmacokinetics of cytostatics, it is currently considered that they should be administered in the same way as in non-pregnant women [113].

Fetal toxicity in monotherapy vs. polychemotherapy shows a slight increase, from 17 to 25% in the case of multiple chemotherapy. Indeed, the antineoplastic effect is more consistent for multiple cytotoxic incidents but with a difficult tolerance on the part of the mother [114].

The risk of use of cytostatics during pregnancy has been classified by the FDA (Food and Drug Administration) into two groups: C and D. Category C suggests that risk cannot be ruled out. There have been no satisfactory studies in pregnant women; however, animal studies have demonstrated a risk to the fetus. The potential benefits of the drug may outweigh the risks. Category D refers to evidence of risk (studies in pregnant women have demonstrated a risk to the fetus; potential benefits of the drug may outweigh the risks) [115,116,117].

These effects caused by the exposure of the fetus to chemotherapy are primarily influenced by the molecular weight of the drugs used and the gestational age (placental structural changes), where both influence the transplacental passage of the cytotoxic agent (Table 2). Other conditions favoring transplacental transfer have also been described, such as dose, ionization at physiological pH, lipophilicity, binding capacity to plasma proteins, and active drug transporters, such as p-glycoprotein and BCRP (breast cancer resistance protein), which could affect the transfer rate. If the passage occurs, it is important that the fetus is as advanced in age as possible for the effects to be minimal.

In general, low-molecular-weight cytostatics (<500 Dalton) that are highly lipophilic and not bound to serum proteins have the ability to cross the placenta. Previous studies have confirmed this in baboons and chimpanzees, which are considered the closest to human genetics and structure, allowing for the selection and recommendation of some chemotherapy drugs [118].

Taxanes and anthracyclines seem to have low rates of transplacental passage, while platinum salts easily penetrate the placental barrier. Alkylans (cyclophosphamide) and antimetabolites (methotrexate) are associated with a high rate of malformations, which is why there is a reluctance to administer them [119].

The second important element in terms of fetal damage is gestational age. All recent studies confirm the risk of administration in the first trimester of pregnancy due to possible congenital malformations in the fetus (approx. 20% of cases), carcinogenesis, organ toxicity, and developmental delay (40%). In the general population, the risk of malformations is between 1 and 3% [114,120,121,122,123]. The important period of organogenesis lasts up to about eight weeks [106,107,114]. Postponing chemotherapy until after this period appears mandatory or at least prudent. Abortion seems to be an acceptable solution if a rapid introduction of chemotherapy is required. The risks of fetal damage decrease with increasing gestational age, which is why there is a much safer approach. Possible benefits to the mother justify the risk of cytostatic treatment. Relatively recent studies have not identified an association between the introduction of chemotherapy and possible teratogenic fetal effects or other complications already presented above (abortion, premature birth, etc.) [124,125,126,127]. Chemotherapy should not be given after week 33, as birth can occur any time after this. The decision to induce labor must take into account the last cytostatic treatment administered; between this and the birth, there must be a free interval of three weeks, avoiding the critical moment of days 7–14 post-chemotherapy. An alternative would be weekly therapy, especially using doxorubicin, paclitaxel, and epirubicin regimens, associated with the minimization of hematological effects in the mother and a much faster recovery for delivery [128,129]. The relative safety of prenatal chemotherapy is mainly known for treatments used in breast cancer, cervical cancer, and lymphomas, while experience with gastric cancer is limited. Most large case series focusing on gastric cancer during pregnancy do not report the use and consequences of cytotoxic treatment and include only Asian patients. The treatment outcomes may present geographical differences [10,19].

The standard cytostatic treatment for primary gastric cancer consists of a combination of platinum and fluoropyrimidine, such as FOLFOX (5-fluorouracil, leucovorin, oxaliplatin), CAPOX (capecitabine, oxaliplatin), ECF/ECC (epirubicin, cisplatin, 5-FU/capecitabine), or EOX (epirubicin, oxaliplatin, capecitabine). Trastuzumab combinations can be given in gastric cancers with the overexpression of HER2. Alternatively, taxane-based regimens such as FLOT (5-FU, leucovorin, oxaliplatin, docetaxel) can be applied [10,130]. The progress of the neoplasia seems to be augmented by estrogen. Estrogen receptors (ERs) are identified in 22% of tumor cells, especially in the poorly differentiated type. Unlike other target organs, e.g., the breast, ERs in gastric cancer appear to be a mark of tumor adaptation. Anti-estrogen treatment is far from a widely accepted therapy and is still under evaluation [17,131,132].

Neoadjuvant chemotherapy is recommended during pregnancy between 10 and 28 weeks for stage II and III tumors. Adjuvant therapy is recommended in addition to surgery, usually after birth [10].

### 7.2. Radiotherapy

Radiotherapy is not an option; it is a solution for other neoplastic sites on the digestive tract, but not for gastric cancer.

### 7.3. Molecular Therapies

Molecular therapies can be useful for the treatment of gastric cancer in pregnancy (Table 3). Targeted therapies are a complex and heterogeneous method in the treatment of gastric cancer, experiencing spectacular development in the last 10–15 years.

An upgrade from the Lauren classification allowed for the introduction of new molecular subtypes of gastric adenocarcinoma, the most well-known being TCGA and ACRG, which have resulted in the identification of evolutionary carcinogenesis mechanisms and subsequently new molecular therapies [134,135,136]. The vast majority of these therapeutic agents are hydrophilic and are characterized by a high molecular mass (although tyrosine kinase inhibitors (TKIs) have low molecular weight); this represents a limitation with respect to transplacental passage.

### 7.4. Anti-HER2 Therapy

Anti-HER2 therapy is based on HER2 protein overexpression and/or gene amplification (HER2 positivity) in gastric cancer. Identification is carried out by immunohistochemistry (IHC) and fluorescence in situ hybridization (FISH) as a mandatory test [137]. About 12–24% of patients are positive with therapeutic indications [138,139,140]. The main agent used is Trastuzumab, which is accepted by the FDA (2010) together with chemotherapeutic agents.

### 7.5. Anti-EGFR Therapy

EGFR activation results in cell proliferation and tumorigenesis. EGFR is identified in about 30% of patients and is considered a favorable prognostic factor [134,141]. Trials using anti-EGFR therapies (Cetuximab and Panitumumab) have reported controversial results, some suggesting a good response rate to treatment while others emphasize the ineffectiveness of this therapy [142,143,144].

### 7.6. Anti-VEGF Therapy

The effect of VEGF is linked to the activation of endothelial proliferation pathways, which are involved in tumor angiogenesis, being identified by overexpression in gastric tumors [145]. The presence of VEGF is an unfavorable prognostic factor, and anti-VEGF therapy is used together with chemotherapy [146,147]. The main monoclonal antibody used is Bevacizumab; however, there is also a newer agent, Ramucirumab. Both are approved to be used in the treatment of advanced gastric cancer in combination with chemotherapy due to good results in research trials [148,149,150,151].

### 7.7. Immunotherapy

Other methods of treatment have been described, perhaps the most interesting being immunotherapy. The discovery of the overexpression of the PD-L1 gene in gastric cancer opened a path for research on the identification of its inhibition factors [152]. The main agent with positive outcomes is Nivolumab (Nivolumab and Ipilimumab), which was approved for use in anticancer therapy in Japan [153]. Pembrolizumab has been approved by the FDA in the USA as a second therapeutic drug in the treatment of gastric cancer [154,155].

### 7.8. Surgical Approach

Surgery in gastric cancer is well defined; however, in the presence of pregnancy, attitudes seem to be particular to each case. On the other hand, the low number of cases and even lower number of consistent works in the literature on this topic (i.e., not just simple case reports) make it difficult to obtain a standard management plan. Ueo [51] published a paper in 1989 that systematizes surgical therapy in pregnancy-associated gastric cancer, where the central element of management is the gestational age. Unfortunately, the paper overlooks the anesthetic and surgical fetal risk, tumor stage, and the mother’s choice. In the last two decades, no reviews have been published that extensively analyze the place of surgery in the treatment of these patients; however, we did identify papers and guidelines that concern anesthesia and surgery during pregnancy [156,157,158,159,160,161,162,163,164].

The therapeutic management needs to be structured according to gestational age, tumor staging, the anesthetic and surgical risks on the fetus, and the mother’s choice.

Up to 22 weeks of pregnancy, in accordance with the recommendations of the Japanese Classification of Gastric Carcinoma, which is identical to the Union for International Cancer Control (UICC)/TNM 8th edition [165,166], for cases with early tumors (T1A), endoscopic resection is the optimal solution that also preserves pregnancy. Unfortunately, such cases are an exception. In the T1B-4N0 range, resection surgery is recommended as soon as possible after abortion. Gastrectomy is carried out according to the TNM staging criteria (Table 4) and tumor topography.

For antropyloric tumors, subtotal resections with lymphatic dissection are standard.

Recently, it has been possible to preserve pregnancy after surgical resection before 22 weeks in cases with relatively early neoplasia [138]. The risk of maternal death is low (<1/10,000) [157,160].

Major abdominal surgery is associated with an increased risk of fetal death and spontaneous abortion in the range of 8–11% but does not induce a risk of fetal malformations. A series of intra- and post-operative complications (e.g., hemorrhage) can affect the fetus via hypoperfusion, hypoxia, and hypotension [160,167,168]. There are few studies in this area; however, an assessment of 2853 pregnant women in their first trimester (out of a total of 12,000) did not show an increase in birth defects. To reduce fetal risk, professional associations recommend adapting the patient’s position on the operating table, intraoperative fetal cardiac index monitoring, regional anesthesia assistance, and adjusting drug doses, inhalant or intravenous, to the lowest possible level [169,170].

Cases with complicated neoplasia (e.g., perforation, hemorrhage) have a bad prognosis for the mother and, consequently, for the fetus. This complication is already a sign of an advanced neoplasia that forces the surgeon to a therapeutic approach to save the mother—a palliative resection. The fetal risk is high, and keeping the pregnancy appears unjustified.

Between 22 and 28 weeks, the anesthetic and surgical risks are lower as organogenesis is completed. If the diagnosis is established at the beginning of the interval, resection can be proposed after inducing delivery, when possible, or resection can be performed with the preservation of the pregnancy [167]. Neoadjuvant therapy is also an option, where the fetal risk is consistently lower than in the case of resection. If the diagnosis is established at the end of the interval, waiting and fetal monitoring until the fetal risk decreases, usually until after 32–34 weeks, followed by delivery and resection, is the optimal choice. In the case of emergencies—complications of neoplasia—the mother’s survival prevails.

After 28 weeks, birth can occur safely in a few weeks. This is why the recommendation is to monitor the patient and the fetus until birth. The delivery can occur naturally or by cesarean section, followed by gastrectomy after approximately 10–14 days. Gastrectomy during cesarean section is not recommended, as the hemorrhagic and thromboembolic risk is significant. For complicated tumors, which require emergency surgery, the induction of labor is mandatory, as the fetus has a high chance of survival [168].

## 8. Prognosis

In a retrospective analysis of clinicopathological characteristics and outcomes in 4722 non-pregnant patients, female gender was significantly associated with a younger age at diagnosis and poorly differentiated adenocarcinoma (signet ring cell carcinoma). Because of these characteristics, overall survival was poorer for women than for men, particularly among patients younger than 45 years of age with advanced disease. The histological features of stomach cancer in pregnancy are similar to those reported in non-pregnant women. However, gastric cancer during pregnancy has a poorer prognosis, with a 3-year overall survival of 23.3% [171,172]. The spreading of tumor cells from mother to fetus is rare. The placental syncytiotrophoblast is the first barrier that protects the fetus from neoplastic invasion, and the invasion of the placental villi implies a high risk of fetal metastases. The second barrier is the fetal immune system. Fetal metastasis of maternal cancer is rare, although it may occur secondary to the immaturity of the fetal immune system. Almost all case reports describing fetal metastases involve the invasion of the placental villi. In cases with maternal malignancy, it is important to evaluate the placenta using multiple sections. If the placenta is involved, the newborn should be carefully evaluated for metastases in the neonatal period and throughout childhood [173].

If fetal metastasis has occurred, it usually becomes evident 4 to 5 months after birth, and the clinical signs can be observed from the time of birth to 20 months after birth. The routine examination of the placenta is mandatory in women with advanced cancer to assess the risk of fetal metastasis. If the placenta is free of metastatic disease, clinicians can reassure parents that the risk of spreading to the fetus is low. Altman et al. suggested that placental metastases should be considered as an indication of stage IV maternal disease and treated accordingly [174]. Mothers should be carefully evaluated in the postpartum period and treated appropriately if placental metastases are identified. A study carried out by Al-Adnani et al. in 2007 considered 75 patients with placental metastases identified between 1930 and 2006, of which 18 had villous invasion [175].

Melanoma was the most common histological type, accounting for 30% of cases of placental metastasis. Fetal metastases were recorded in 11 cases (in 50% of the cases, it was also melanoma) [176].

A recent study that compared overall survival in 20 patients with pregnancy-associated gastric cancer aged 39 years with similar stages and age to non-pregnant women concluded that advanced stage and tumor location are poor prognostic factors regardless of pregnancy status [20].

Perhaps the most important aspect compared to the general population is the incidence of aggressive tumors, with poor, undifferentiated tumor grading (macroscopically infiltrative type, 84%; diffuse type, 87%) [19].

If we add to this the advanced stage of gastric tumors in pregnant women (95%) and the presence of pregnancy, which makes therapy even more difficult, we can understand why the prognosis is generally poor. According to a Japanese study, resection is only possible in 45% of cases, which also includes palliative resections. Evaluating only cases from the last 20 years did not lead to better results. Long-term survival confirmed the poor prognosis: 18% at 1 year, 15% at 2 years, and only one patient alive at 3 years. Regarding fetal prognosis, three aspects are identified: risk of neoplastic invasion, risk of premature birth, and risk of fetal death. Fetoplacental metastases are rare; only three cases have been reported, two placental metastases and one case of fetal metastasis [177].

Regarding fetal survival, statistical data on 92 patients, regardless of the initial gestational age at the time of diagnosis, show a relatively favorable prognosis, with 77% of newborns surviving the medical event. If the gestational age is greater than 30 weeks, the survival rate approaches 100%. Fetal death or the risk of miscarriage are possible events at any time during therapy for gastric cancer. The most sensitive interval is before 28 weeks. More recent data revealed that following resection therapy, the pregnancy continued to progress in 21% of cases [43].

### Prognosis Markers in Pregnancy-Associated Gastric Cancer

Pregnancy-specific glycoproteins (PSGs) belong to the immunoglobulin superfamily and contain four types of immunoglobulins (Ig) [178].

There are 10 genes (PSG1-9, PSG11) encoding human PSGs. PSG is a placental protein mainly expressed during pregnancy that is known to play roles in immune regulation, angiogenesis, and platelet function [179].

Among them, pregnancy-specific beta-1-glycoprotein 1 (PSG1, known as SP1) is a pregnancy-associated glycoprotein mainly expressed in the placenta. It is thought to play a role in various processes, including implantation, trophoblast differentiation, and the activation of angiogenesis [180].

PSG1 is related to TGF-β and mainly regulates the vascular endothelial growth factor family (VEGF A, B, C, and D) and placental growth factor (PGF) [181].

The abnormal expression of PSG1 does not allow a normal pregnancy. PSG1 is associated with carcinoembryonic antigen (CEA) and has been reported to be expressed in various cancers [182].

In particular, PSG1 is increased in gastric cancer and is used as a biomarker for the diagnosis of this neoplasia. PSG1 is a type of protein that is secreted, as opposed to the common membrane-attached protein CEA. This feature allows for its detection in the patient’s blood, which is one of the essential requirements for a diagnostic marker. However, further studies are needed to determine the potential of PSG1 as a diagnostic biomarker. Public databases have shown that PSG1 is highly expressed in gastric cancer, yet the role of PSG1 in oncology is not well understood [183].

## 9. Conclusions

In summary, gastric cancer during pregnancy is a rare diagnosis. Women usually present at an advanced stage and have a poor prognosis. The early recognition of symptoms is indispensable for diagnosis at a curative stage. In pregnant women with persistent gastrointestinal symptoms that cannot be explained by pregnancy alone, there should be a low threshold for further diagnostic procedures. While balancing maternal and fetal risks, the initiation of chemotherapy during pregnancy may be considered in order to reach fetal maturity. A multidisciplinary approach is necessary for appropriate decision-making in this difficult and rare situation.

The features and prognosis of stomach carcinoma associated with pregnancy are the same as in other young patients. Therefore, the diagnosis of gastric cancers should be asked for in all patients, not only during pregnancy. In the case of gastric cancer in early pregnancy, abortion must be discussed to allow for optimal treatment. Beyond 24 weeks, the pregnancy should be allowed to continue until the viability of the fetus can be assumed. Then, a cesarean section and the operative treatment of the cancer can be performed. As a consequence of the generally poor prognosis of gastric cancer, efforts must be made to create programs for primary prevention, for example, regarding nutritional habits, quitting smoking, or the eradication of HP in high-risk patients.

## Figures and Tables

**Table 2:** Risks of using chemotherapy during pregnancy.

Pregnancy Period	Implantation Period	Organogenesis Period	Fetal Development Period
Trimester	1st trimester	2nd trimester	3rd trimester
Week	1 2 3 4 5 6 7 8 9 10 11 12 16 24 32 40		
Chemotherapeutic agents	Major congenital malformations Fetal demise Impaired organ function Spontaneous abortion	No definite associations with significant teratogenic effects (limited data)	Minor associations with: - Prematurity - Intrauterine growth restriction - Low birth weight
Targeted agents	Relatively low risk	Stillbirth Lung disease Renal failure Respiratory distress syndrome Severe pulmonary hypoplasia Prematurity	Oligohydramnios/anhydramnios (most common adverse event)
Tyrosine kinase inhibitors TKIs	Avoid imatinib use in the first trimester: - Spontaneous abortion - Major malformations - Embryonic developmental disorders	Teratogenic potential and major malformations: exencephaly, encephalopathies, and abnormalities in the skull bones	Severe maternal adverse effects
IgG4 antibodies	Spontaneous abortion Intrauterine growth restriction Congenital hypothyroidism	No increase in the rate of malformations	Miscarriage, stillbirth, prematurity, small birth weight, and infant mortality

**Table 3:** Cancer-targeted therapies used for gastrointestinal cancer [133].

Chemotherapeutic Agents	Studies on Humans	Studies on Animals	Pregnancy Category *
Bevacizumab	ND	T	C
Cetuximab	ND	T	C
Panitumumab	ND	T*	C
Sorafenib	ND	T	D
Imatinib	ND	T	D

T—teratogenic; T*—data suggesting teratogenicity is not strong enough; ND—no data; Pregnancy category *—U.S. Food and Drug Administration (FDA).

**Table 4:** Tumor stage TNM and surgical recommendation.

TNM Stage	T1bN0	T1N+	T2-4N0	T2-4N+M+
Types of gastric surgery	Gastrectomy D1/D1+	Gastrectomy D2	Gastrectomy D2	Palliative surgery Reduction surgery

## Data Availability

The data presented in this study are available on request from the corresponding author.
